# Role of PsnWRKY70 in Regulatory Network Response to Infection with *Alternaria alternata* (Fr.) Keissl in *Populus*

**DOI:** 10.3390/ijms23147537

**Published:** 2022-07-07

**Authors:** Wei Wang, Xiang-Dong Bai, Kun Chen, Chen-Rui Gu, Qi-Bin Yu, Jing Jiang, Gui-Feng Liu

**Affiliations:** 1 State Key Laboratory of Tree Genetics and Breeding, Northeast Forestry University, 26 Hexing Road, Harbin 150040, China; 15146074137@163.com (W.W.); bxd1207235923@163.com (X.-D.B.); ck15895588@163.com (K.C.); guchenrui@outlook.com (C.-R.G.); jiangjing1960@126.com (J.J.); 2 Citrus Research and Education Center, University of Florida, Lake Alfred, FL 33850, USA; qibin@ufl.edu

**Keywords:** poplar, leaf blight, WRKY, disease resistance, plant immunity

## Abstract

WRKY is an important complex family of transcription factors involved in plant immune responses. Among them, *WRKY70* plays an important role in the process of the plant defense response to the invasion of pathogens. However, the defense mechanism of *PsnWRKY70* is not clear in *Populus nigra*. In this study, we showed that *PsnWRKY70*-overexpression lines (OE) had fewer leaf blight symptoms than *PsnWRKY70*-repressing lines (RE). *PsnWRKY70* activated MAP kinase cascade genes (*PsnM2K4*, *PsnMPK3*, *PsnM3K18*), calcium channel proteins-related genes (*PsnCNG3*, *PsnCNGC1*, *PsnCNG4*), and calcium-dependent protein kinases genes (*PsnCDPKL*, *PsnCDPKW*, *PsnCDPKS*, *PsnCDPKQ*). Furthermore, 129 genes of *PsnWRKY70* putative genome-wide direct targets (DTGs) were identified by using transcriptome (RNA-seq) and DNA affinity purification sequencing (DAP-seq). *PsnWRKY70* directly binds to the promoters of homologous genes and LRR domain proteins to promote the expression of *WRKY6*, *WRKY18*, *WRKY22*, and *WRKY22–1*, LRR domain proteins *LRR8*, *LRR-RLK*, *ADR1-like 2*, *NB-ARC*, etc. Our study suggests that *PsnWRKY70* enhances the resistance of *A. alternata* in poplar by activating genes in both pathogen-associated molecular pattern-triggered immunity (PTI) and effector-triggered immunity (ETI).

## 1. Introduction

In the natural environment, plants are constantly confronted with diverse biotic and abiotic stresses that adversely affect their growth and development [1]. Pathogen attack is one of the most limiting factors affecting plant growth and thus poses a serious threat to the agroforestry industry worldwide [1,2,3]. *Alternaria alternata* is a necrotrophic fungal pathogen. Its multiple pathological races can infect many plants, such as chrysanthemum (*Chrysanthemum morifolium*), tobacco (*Nicotiana attenuata*), and poplar (*Populus. tomentosa*), causing huge national economic losses [3,4,5].

Plants have developed multitiered defense mechanisms for protection against pathogens. Plants have evolved pathogen-associated molecular patterns (PAMP)-triggered immunity (PTI) and effector-triggered immunity (ETI) systems [6,7]. Usually, a reactive oxygen species (ROS) burst occurs by binding to cell membrane surface co-receptors and by phosphorylating RLCKs, MAPKs, and calcineurin kinases [8,9,10,11]. At the same time, intracellular nucleotide-binding LRR (NLR) receptors cooperate with lipase-like proteins such as *EDS1*, *SAG101*, and *PAD4* to induce ETI through NLRs of the ADR1 family and the NRG1 family [12]. In *N. benthamiana*, the MAPK cascade activates *WRKY7*, *WRKY8*, *WRKY9,* and *WRKY11*. These WRKYs can bind to the W-box element in the *RBOHB* promoter to upregulate the *RBOHB* gene and cause a ROS burst [13]. Recent studies have shown that, in *Arabidopsis*, activation of the *RPS2* gene leads to the accumulation of BIK1 and RBOHD proteins and increased transcription of *WRKY29* and *AZI* [14]. Pathogen defense response is achieved through large-scale transcriptional activation of defense-related genes [15,16]. WRKY transcription factors play a crucial role in the regulatory network for defense against pathogenic infection [17]. WRKYs are mainly characterized by a highly conserved WRKYGQK motif and a zinc finger motif [18]. WRKYs exert their regulatory functions by binding to the DNA sequence of the W-box (TTGACT/C) [19,20]. *PtrWRKY89* accelerates pathogenesis-related protein (PR) gene expressions and enhances pathogen resistance in transgenic poplar (*Populus trichocarpa*) and is a regulator of salicylic acid (SA)-dependent defense signaling in poplar [21]. Liu et al. identified *BnWRKY33* as a *S. sclerotiorum* responsive gene by enhancing the expression of genes involved in camalexin synthesis and regulating SA and jasmonic acid (JA) to defend against the pathogen [22].

We previously showed that *PsnWRKY70* overexpression improved leaf blight resistance in *Populus*. Fifteen days after *A. alternata* infection, the leaf disease index and MDA content of OE lines were significantly lower than those of WT lines, and the plant height was significantly higher than that of WT lines [23]. However, little is known about the defense mechanism of how poplar trees respond to the infection of *A. alternata*. The objective of the study was to investigate the defense mechanism of *PsnWRKY70* and understand its role in the regulatory network response to infection with *A. alternata*. We performed RNA-seq analysis to investigate differentially expressed genes regulated by *PsnWRKY70* during disease resistance in poplar. We also identified direct genome-wide target genes of *PsnWRKY70* using DAP-seq. The key direct target genes regulated by *PsnWRKY70* were verified in vivo by CHIP-PCR and qRT-PCR. Our results provide new insight into the molecular mechanism of the *PsnWRKY70* transcription factor in forest disease resistance and could be used as one technical guidance for molecular breeding of forest disease prevention.

## 2. Results

### 2.1. PsnWRKY70 Reduces Infection with A. alternata

**Figure 1:**
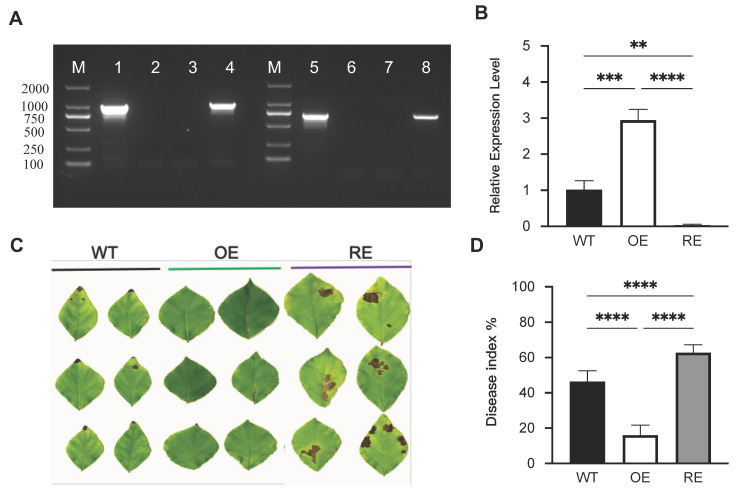
Molecular detection and disease severity of *PsnWRKY70* transgenic lines infected with *A. alternata*. (**A**) PCR amplification of *PsnWRKY70* transgenic lines, DNA ladder DL2000 (lane M), positive control (pGWB5-*PsnWRKY70*-GFP plasmid) (lane 1), water control (lane 2), WT (lane 3), *PsnWRKY70* OE lines (lane 4), positive control (pGWB5-amiRNAi*PsnWRKY70*-GFP plasmid) (lane 5), water control (lane 6), WT (lane 7), and *PsnWRKY70* RE lines (lane 8). (**B**) qRT-PCR detection of *PsnWRKY70* transgenic lines (*n* = 3, ** *p* < 0.01, *** *p* < 0.001, **** *p* < 0.0001). (**C**) Phenotype characterization of each transgenic line with inoculation of *A. alternaria* for 15 days. (**D**) Disease index of each transgenic line with inoculation of *A. alternata* after 15 days (*n* = 9, **** *p* < 0.0001).

To ensure the accuracy of gene transformation, PCR molecular detection was performed on the *PsnWRKY70*-transformed poplars (Figure 1A). At the same time, qRT-PCR results show that compared with the WT line, the gene expression of *PsnWRKY70* was significantly upregulated, 2.94 times in OE compared to that of WT (Figure 1B); on the contrary, the relative expression of *PsnWRKY70* in the RE was significantly lower than that in the WT (Figure 1B). All the tested lines were susceptible to *A. alternata* in different degrees after 15 days of inoculation (Figure 1C,D). The leaves of the OE line had fewer disease spots and more green leaf color. OE had a 16% disease index in comparison with 46.3% of the WT line. The RE line had a 62.8% disease index and leaves showed chlorosis and yellowing with serious disease.

### 2.2. Transcriptime Analysis

**Figure 2:**
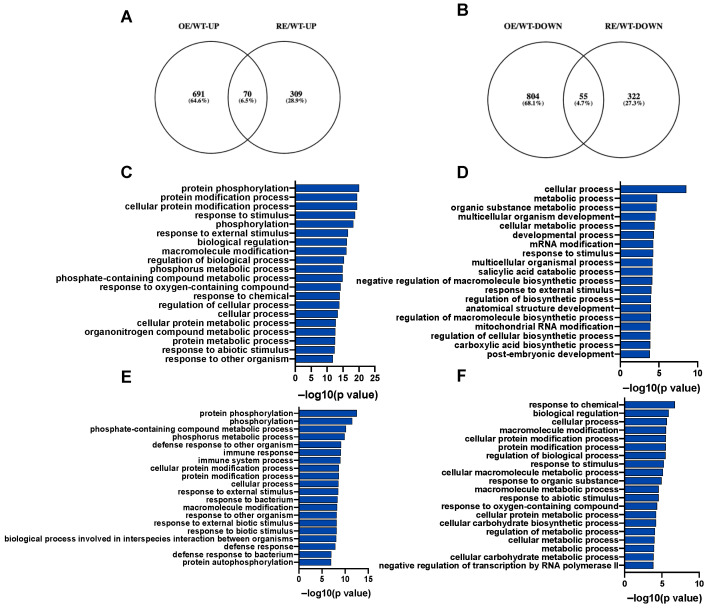
Transcriptome analysis of *PsnWRKY70* transgenic lines infected with *A. alternata*. (**A**) Venn diagram of upregulated DEGs between OE and RE. (**B**) Venn diagram of downregulated DEGs between OE and RE. (**C**) GO enrichment analysis of uniquely upregulated genes in OE lines. (**D**) GO enrichment analysis of uniquely downregulated genes in OE lines. (**E**) GO enrichment analysis of uniquely upregulated genes in RE lines. (**F**) GO enrichment analysis of uniquely downregulated genes in RE lines.

**Figure 3:**
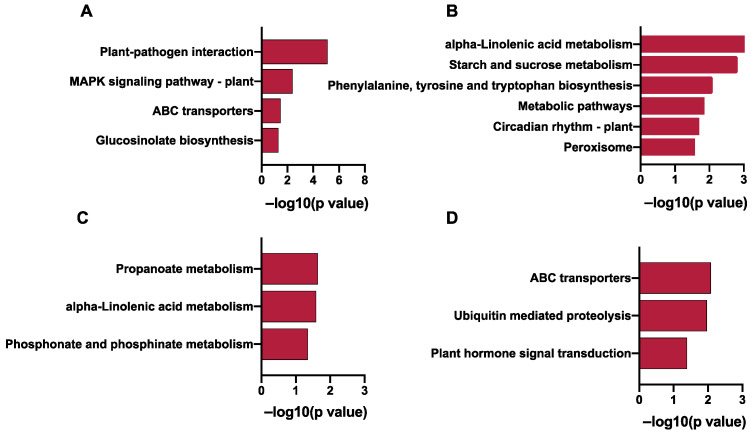
KEGG enrichment analysis of differential genes in *PsnWRKY70* transgenic poplar. (**A**) KEGG enrichment analysis of uniquely upregulated genes in OE lines. (**B**) KEGG enrichment analysis of uniquely downregulated genes in OE lines. (**C**) KEGG enrichment analysis of uniquely upregulated genes in RE lines. (**D**) KEGG enrichment analysis of uniquely downregulated genes in OE lines.

A total of about 86 Gb of clean data was obtained with an average of about 9.5 Gb per sample. The mapping rate was between 81.72% and 84.68% (Table S2). The thresholds of DEGs between OE vs. WT and RE vs. WT were used with fold change ≥ 2 and *p*-value < 0.05. There were 691 and 309 exclusively upregulated DEGs in OE and RE lines. There were 70 overlap DEGs between OE and RE (Figure 2A). There were 804 and 322 exclusively downregulated DEGs in OE and RE lines. There were 55 overlap DEGs between OE and RE (Figure 2B). GO analysis showed that the 691 upregulated DEGs in OE were enriched in protein phosphorylation, cellular protein modification process, and response to stimulus (Figure 2C). The 309 upregulated DEGs for RE were enriched in protein phosphorylation, defense response to other organism, and immune response (Figure 2E). The 804 downregulated DEG for OE were enriched in cellular process, metabolic process, and organic substance metabolic process (Figure 2D). The 322 downregulated DEGs for RE were enriched in response to chemical, biological regulation, and cellular process (Figure 2F). KEGG enrichment analysis showed that 691 DEGs upregulated for OE were enriched in plant–pathogen interaction, MAPK signaling pathway–plant, and ABC transporters (Figure 3A). The 309 upregulated DEGs for RE were enriched in propanoate metabolism, alpha-Linolenic acid metabolism, and phosphonate and phosphinate metabolism (Figure 3C). The 804 downregulated DEGs for OE were enriched in alpha-Linolenic acid metabolism, starch and sucrose metabolism, phenylalanine, tyrosine, and tryptophan biosynthesis (Figure 3B). The 322 downregulated DEGs for RE lines were enriched in ABC transporters, ubiquitin-mediated proteolysis, and plant hormone signal transduction (Figure 3D).

### 2.3. PsnWRKY70 Activates the Expression of MAPK and Ca2^+^ Signaling-Related Genes

**Figure 4:**
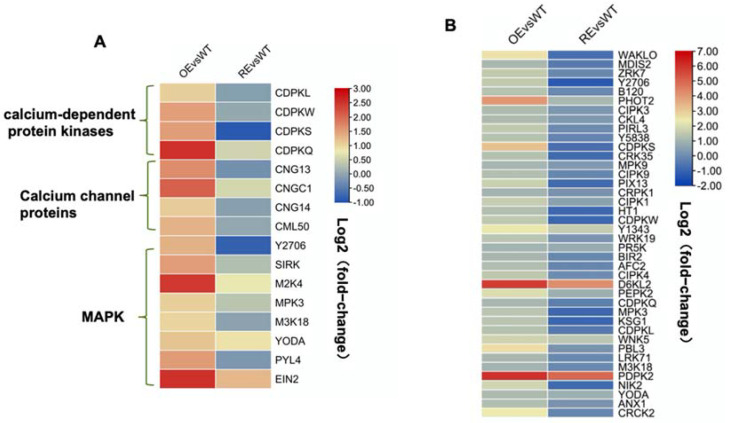
Expression of immune-related genes in *PsnWRKY70* transgenic line. (**A**) KEGG enriched genes in the plant–pathogen interaction pathway. (**B**) GO enriched genes in the protein phosphorylation pathway.

**Figure 5:**
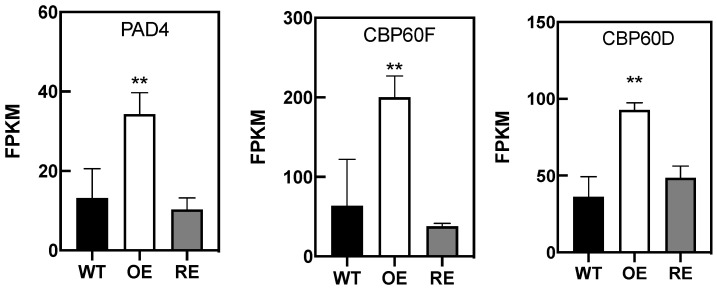
Expression of SA-related genes in *PsnWRKY70* transgenic poplar (*n* = 3, ** *p* < 0.01).

Genes that play important roles in disease resistance were regulated by *PsnWRKY70* (Figure 4A,B). After OE lines were infected with *A. alternata*, genes in the MAPK cascades, such as *M2K4*, *MPK3,* and *M3K18*, were significantly upregulated. Similarly, Ca2^+^ channel proteins *CNG3*, *CNGC1*, and *CNG4*, and calcium-dependent protein kinases *CDPKL*, *CDPKW*, *CDPKS,* and *CDPKQ* were significantly upregulated as well. In addition, transmembrane receptor protein-related genes *NIK2*, *CRK35*, *CRPK1*, *LRK10L-1.2*, *CRCK2*, *CES101,* and *CRK23* were significantly upregulated. As a result, *PsnWRKY70* enhances disease resistance by upregulating the expression of a MAP kinase cascade, calcium signal-related genes, membrane receptors, and other related genes. SA-related biological processes were enriched with upregulated DEGs in OE (Figure 5). They were *PAD4*, *CBP60C*, and *CBP60D*, but they were not significantly changed in RE.

### 2.4. Genome-Wide Binding Targets of PsnWRKY70

**Figure 6:**
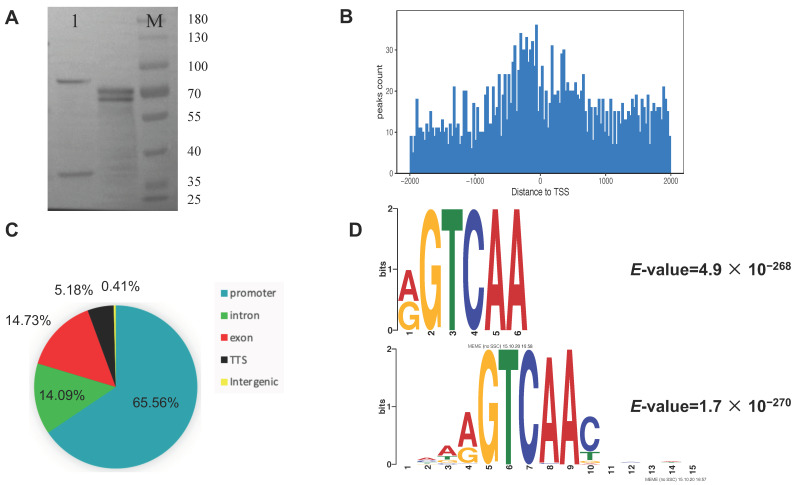
Expression of genome-wide identification of *PsnWRKY70*-binding sites. (**A**) Western blot detection of the expression of *PsnWRKY70* fusion protein. (**B**) Genomic distribution of *PsnWRKY70*-binding peaks. (**C**) Distribution of *PsnWRKY70*-binding peaks relative to gene structure. (**D**) Motif analysis of the *PsnWRKY70* peak revealed the W-box as the highest enriched motif.

The target protein of *PsnWRKY70* was expressed by the wheat germ protein expression system and the protein expression was detected by Western blot. The detection results are shown in Figure 6A: the fusion protein is about 70 kD. After filtering the original DAP-seq data, a total of about 2.95 Gb of data was obtained, including 5.6~6.5 million clean reads per sample (Q30 ≥ 89.82%) (Table S3). The mapping rate of the unique locus of the gene was 40.38–42.24%. The total mapping rate (including the multi-locus alignment of the genome) was 71.05–73.92% (Table S4). The 1 kb flanking sequences around the peaks of all genes were applied to the motif discovery tool MEME-CHIP to explore the *PsnWRKY70* transcription factor binding motif (Figure 6D). The results showed that the main motifs bound by *PsnWRKY70* were “NMAAGTCAACNNNDN“ (E-value = 1.7 × 10^−270^) and “RGTCAAY” (E-value = 4.9 × 10^−268^). *PsnWRKY70* peaks in DAP-seq were distributed over gene regions. The transcription starting site is 2 kb downstream of the transcription termination. *PsnWRKY70* binding sites were identified in 2103 gene regions. Among them, 65.56% were in the promoter region (−2 kb to +500 bp of TSS), 0.41% in the intergenic region, 14.09% in the intron region, 14.73% in the exon region, and 5.18% in the terminator region (Figure 6C). The identification promoter site for *PsnWRKY70* was based on the distance between each peak and its closest gene TSS. It was determined that the *PsnWRKY70* binding site was significantly concentrated at 300 bp upstream of TSS in the core promoter region (Figure 6B). These distribution patterns of *PsnWRKY70* binding sites in DAP-seq experiments are consistent with the fact that *PsnWRKY70* acts as a transcription factor.

### 2.5. Target Genes Were Regulated by PsnWRKY70

**Figure 7:**
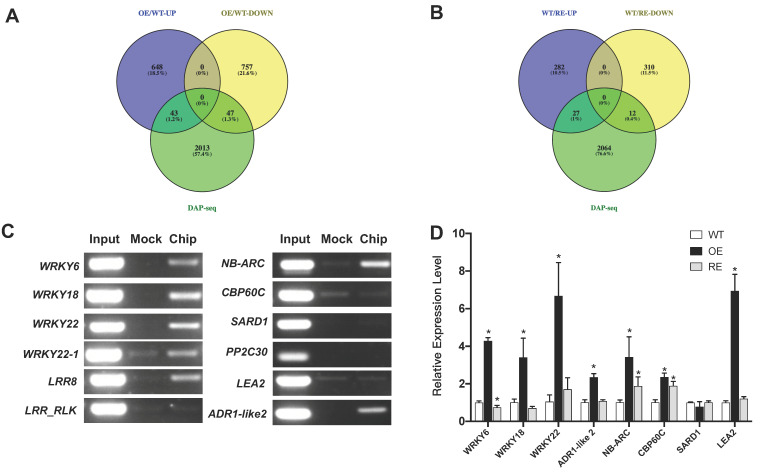
Validation of downstream target genes of *PsnWRKY70* gene. (**A**) Venn diagram of upregulated genes between RNA-seq and DAP-seq. (**B**) Venn diagram of downregulated genes between RNA-seq and DAP-seq. (**C**) CHIP-PCR for downstream genes of *PsnWRKY70*. (**D**) qRT-PCR of downstream genes of *PsnWRKY70* (*n* = 3, * *p* < 0.05).

To find the downstream target genes directly regulated by *PsnWRKY70*, the Venn map showed that *PsnWRKY70* directly regulated 43 target genes among 691 upregulated genes in OE lines (Figure 7A). These include the WRKY family homologous genes *WRKY6*, *WRKY18*, *WRKY22*, and *WRKY22-1*. These WRKY family homologous genes play an important role in the process of resistance to pathogenic bacteria. It also includes some R genes that play an important role in disease resistance, such as leucine-rich repeat (*LRR8*), leucine-rich repeat receptor-like protein kinase family protein (*LRR-RLK*), *ADR1-like 2,* similar to putative disease resistance protein, LRR protein, *NB-ARC* domain, and LRR-containing proteins (Table 1). Among the 322 downregulated genes in RE lines, *PsnWRKY70* directly regulated 12 target genes (Figure 7B). These include LOB domain-containing protein 41, *LRR-1*, photosystem II subunit R, glycosyl transferase family 2 protein, and some others. These genes positively regulated by *PsnWRKY70* play an important role in disease resistance. Similarly, among the 804 downregulated genes in the OE lines, *PsnWRKY70* directly targeted 47 of them (Figure 7A). Among the 309 upregulated genes in RE lines, *PsnWRKY70* directly regulated 27 of them as target genes (Figure 7B). These *PsnWRKY70* negatively regulated genes may also play a role in disease resistance. qPCR validation showed that *WRKY6*, *WRKY18*, *WRKY22*, *ADR1-like2*, *CBP60C*, and *LEA2* genes were significantly upregulated in OE lines (*p* < 0.05) (Figure 7D). In addition, CHIP-PCR showed that *WRKY6*, *WRKY18*, *WRKY22*, *WRKY22-1*, *ADR1-like2*, *NB-ARC,* and *LRR8* amplify clear bands. This suggests that *PsnWRKY70* directly regulates *WRKY6*, *PsnWRKY18*, *WRKY22*, *WRKY22-1*, *ADR1-like2*, *NB-ARC,* and *LRR8*, thereby enhancing the resistance to *A. alternata* (Figure 7C).

## 3. Discussion

### 3.1. PsnWRKY70 Induces Other WRKYs to Enhance Disease Resistance

WRKYs typically contain functional W-boxes in their promoters and can be regulated by their own gene products or other WRKYs [24,25,26,27]. In Parsley (*Petroselinum crispum*), *PcWRKY1* binds to the W-box element on its own promoter for self-regulation, and can also bind to the W-box element on the promoter of *PcWRKY3* [25]. In *Arabidopsis*, WRKY46 interacts with NPR1 to bind to the W-box of the *WRKY6* promoter to induce its expression in response to SA signaling. Overexpression of *WRKY6* is sufficient to accelerate leaf senescence [26]. In Rice (*Oryza sativa*), *WRKY45*-*2*, *WRKY13,* and *WRKY42* form a transcriptional regulatory cascade in response to *Magnaporthe oryzae* [27]. *WRKY* transcription factor plays an important role in disease resistance. Previous studies have shown that *OsWRKY6* directly binds to WLE1 and W-box in defense-related gene promoters and regulates pathogen defense responses. *OsWRKY6* enhances SA accumulation by activating rice *OsICS1* gene expression involved in SA biosynthesis [15]. *AtWRKY18* enhances PR gene expression and resistance to *Pseudomonas syringae*. However, excessive expression of *AtWRKY18* also leads to severe abnormalities in plant growth [28]. Our data suggest that the *PsnWRKY70* directly binds to the promoters of the homologous genes *WRKY6*, *WRKY18*, *WRKY22*, and *WRKY22*-*1* to induce their expression after infection with *A. alternata*, thereby enhancing the resistance of poplar to necrotrophic fungi invasion.

### 3.2. PsnWRKY70 Plays a Key Role in PTI and ETI Pathways

**Figure 8:**
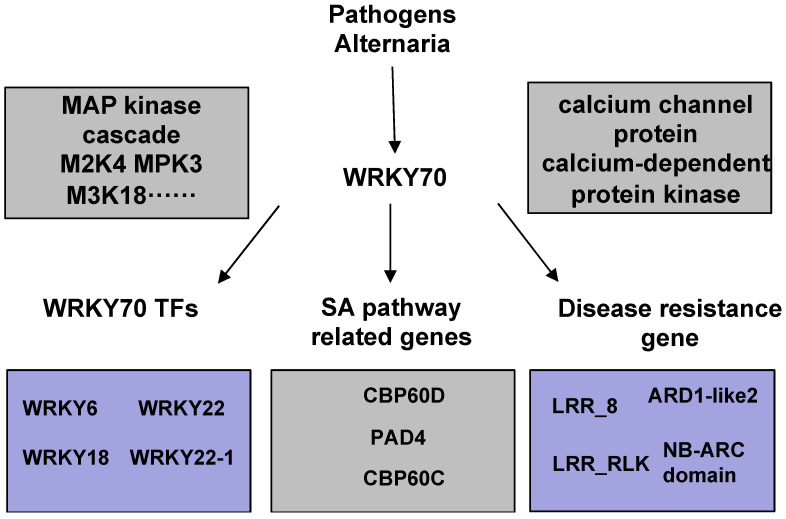
A proposed model of the *PsnWRKY70*-mediated regulatory network response to *Alternaria* infection in *Populus*. *PsnWRKY70* indirectly activated PTI pathway genes, including MAP kinase cascades, calcium channel proteins, calcium-dependent protein kinases, and SA pathway-related genes. It directly binds to the promoters of homologous genes and induces the expression of *PsnWRKY6*, *PsnWRKY18*, *PsnWRKY22*, *PsnWRKY22-1*, and *R* genes in the ETI pathway. The gray represents that *PsnWRKY70* indirectly regulates genes. The purple represents that *PsnWRKY70* directly regulates genes.

Plant immunity has evolved into a complex, multilayered, and sophisticated system to fight against the threat of pathogenic microorganisms [29]. It is well-known that the plant’s innate immune system mainly relies on two major pathogen recognition mechanisms: PTI and ETI [30]. In *Arabidopsis*, the EDS1–PAD4–ADR1 node is a convergence point for defense signaling cascades activated by both surface-resident and intracellular LRR receptors in conferring pathogen immunity through PTI and ETI [31]. Additionally, (Ca2^+^) signaling plays an important role in plant PTI and ETI processes [32]. *OsCNGC9* enhances rice resistance to *Magnaporthe oryzae* by regulating extracellular Ca2^+^ influx to promote ROS burst and activate the expression of PTI-related genes [33]. The formation of calcium channels by *CNGC2* and *CNGC4* is phosphorylated and activated by BIK1. BIK1 triggers an increase in cytosolic calcium concentration, which in turn activates immune signaling [34]. SA is a phytohormone essential for signaling in the pattern PTI and ETI [35]. SARD1 is a plant immune activator that promotes the production of the hormone SA and the activation of defense gene expression [36,37]. In the uninfected stage, *AtWRKY70* represses the expression by binding to the GACTTTT motif on the *SARD* gene promoter and indirectly repressing *CBP60g* and *SID2*. In the infected stage, *CBP60g* and *SARD1* are significantly induced. ES4326, *CBP60g,* and *SARD1* are significantly induced by *Pseudomonas syringae pv. Maculicola* [38]. In Figure 8, we show that the *PsnWRKY70* gene acts as a regulator to mediate the activation of PTI and ETI pathways of innate immunity. Our study found that *M2K4*, *MPK3*, and *M3K18* in the MAPK cascade pathway were significantly upregulated in OE lines, along with calcium channel protein genes *CNG3*, *CNGC1*, and *CNG4*, and calcium-dependent protein kinase (*CPKs*) genes *CDPKL*, *CDPKW*, *CDPKS*, and *CDPKQ*. In addition, it was also found that SA biosynthesis-related genes *PAD4*, *CBP60C*, and *CBP60D* were also significantly upregulated in OE lines. The overexpression of the *PsnWRKY70* gene in the OE line upregulated the expression of the MAP kinase cascade, calcium signal-related genes, SA synthesis genes, membrane receptors, and some other genes, thereby enhancing the resistance to *A. alternata*.

## 4. Materials and Methods

### 4.1. Plant Material

Transgenic plants (*Populus simonii* × *Populus nigra*) of *PsnWRKY70* used in this study were described previously [23]. They are the overexpression line (OE), the repressed expression line (RE), and the wildtype (WT). Plants were grown in the Breeding Base of Northeast Forestry University in Harbin, Heilongjiang Province (126°64′ E, 45°72′ N). In the greenhouse, 15 cm-long stem cuttings were propagated in a substrate of peat soil:river sand:black soil (*v/v*) = 2:2:1 (16:8 h, light:dark, 22:25 °C) for 30 days. For each transgenic line, 9 plants with the same growth status were selected for treatment. To ensure the accuracy of transgenic plants, PCR molecular detection was performed on the *PsnWRKY70* gene-transformed poplar. The PsnW70-F/PsnW70-R and A/B primer pairs were used for PCR validation of OE and RE. Primers are listed in Table S1.

### 4.2. Evaluation of Disease Severity

Plants for OE, RE, and WT lines were inoculated by spraying with *A. alternata* spore suspension (5.0 × 10^7^ spores mL^−1^). Plants sprayed with water were used as controls for OE, RE, and WT lines. Each treatment had three biological replications. Disease severity scores were recorded and photographed after inoculation for 15 days. The disease index was calculated using the method [23]. The leaves were scored on a scale from 0 to 4: 0 (healthy leaves), 1 (<20% of leaves with spots), 2 (about 50% of leaves with spots), 3 (>80% of leaves with spots), and 4 (leaf is dead). Leaf disease index = ∑ (i × Ni) (Nt × Gmax)/ (Nt ×Gmax) × 100%, where i is the grade level (i.e., 0–4), Ni is the number of grades of i leaves, Nt is the total number of leaves, and Gmax is the highest grade level (i.e., 4) [23].

### 4.3. Transcriptome Analysis

Total RNA was extracted from the whole leaves of WT, OE, and RE transgenic plants using a universal plant total RNA extraction kit (BioTeke Corporation, Beijing, China) after *A. alternata* infection for 15 days. RNA samples were submitted for 150 bp paired−end reads on Illumina × 10 platform. To ensure the quality of the analysis data, the original sequences were filtered to obtain high-quality clean reads. Clean reads were mapped to the *Populus trichocarpa* genome using hisat2 [39]. The mapped reads were counted using Stringtie software [40]. Significance analysis of differentially expressed genes (DEGs) was performed using DEseq2 (*p* < 0.05, fold change ≥ 2) [41]. Each treatment sample had three sequencing biological replicates. DEGs were used for gene ontology (GO) enrichment analysis using the GO enrichment tool (http://geneontology.org (accessed on 1 February 2022)), with a *p*-value less than 0.01 as the threshold for significant enrichment [42,43,44]. DEGs were used for KEGG enrichment analysis using the KEGG enrichment tool (http://kobas.cbi.pku.edu.cn/genelist/ (accessed on 1 February 2022)), with a *p*-value less than 0.05 as the threshold for significant enrichment [45].

### 4.4. Quantitative Real-Time Polymerase Chain Reaction (qPCR)

Total RNA was extracted using a universal plant total RNA extraction kit (BioTeke Corporation, Beijing, China), and cDNA was separately synthesized using the Toyobo Reverse Transcription Kit (ReverTra Ace^®^ qPCR RT Master Mix with gDNA Remover (Toyobo, Osaka, Japan)). qRT-PCR was performed on an ABI-7500 quantitative PCR instrument using Toyobo SYBR^®^ Green Real-time PCR Master Mix Plus (Toyobo, Osaka, Japan). The qPCR data were analyzed by the 2^−ΔΔCT^ method, and 18S was used as the internal reference gene [46]. Primers of target genes are listed in Table S1.

### 4.5. DAP-Seq Analysis

The DAP experiments were performed following the published protocol [47]. Briefly, the genomic DNA of WT leaves was extracted by the CTAB method [48], and the genomic DNA was fragmented about 100–400 bp by Covaris M220, and then the genomic DNA library was prepared. In parallel, the coding sequence of *PsnWRKY70* was cloned into the pFN19K HaloTag T7 SP6 Flexi expression vector. The Halo-*PsnWRKY70* fusion protein was expressed using a TNT SP6-coupled wheat germ extraction system (Promega, USA) in 50 µL reactions and incubated at 37 °C for 2 h. The proteins were captured directly using Magne Halo Tag Beads (Promega, Madison, WI, USA). Finally, the purified protein was incubated with the genomic DNA library. The non-specifically bound DNA was eluted. The DNA fragments that could bind to the *PsnWRKY70* protein were eluted. The library was amplified with primers containing sample-specific barcodes. A sample with two biological replicates for *PsnWRKY70* DAP was used for 150 bp paired-end sequencing on the Hiseq X Ten platform (Illumina, San Diego, CA, USA). Reads were mapped to the *Populus trichocarpa* genome sequence using bowtie2 [49]. Peak calling was performed using Macs2 [50]. Motif discovery was performed using MEME [51]. Associations of DAP-seq peaks located upstream or downstream of the transcription start site (TSS) within 3.5 kb were analyzed using Homer [52].

### 4.6. CHIP-PCR

The CHIP procedure followed the method in [53]. The leaves of OE were cross-linked in a vacuum with a cross-linking buffer containing 1% formaldehyde on ice for 15 min. Chromatin was sonicated into fragments between 300 and 500 bp. The supernatant was incubated with an anti-GFP antibody (Abcam, ab290). Immunoprecipitated DNA fragments were purified using the Qiaquick PCR purification kit (Qiagen, Hilden, Germany). CHIP-PCR was conducted using the immunoprecipitated DNA fragments that were obtained above, and the sheared genome DNA was used as a positive control (primers are listed in Table S1) [54].

### 4.7. Statistical Analysis

Statistical significance was assessed by Student’s t-test and one-way ANOVA (**** *p* < 0.0001, *** *p* < 0.001, ** *p* < 0.01, * *p* < 0.05). Each sample had three biological replicates. The data mean was shown as the mean ± standard deviation (SD).

## 5. Conclusions

Here, we reported previously unknown regulatory pathways that mediate the response of *Populus nigra* to resistance to *A. alternata* necrotrophic fungi. RNA-seq analysis showed that the *PsnWRKY70* gene enhanced the disease resistance by upregulating the expression of the MAP kinase cascade, calcium ion signal-related genes, membrane receptors, and other related genes. In addition, 129 downstream target genes of *PsnWRKY70* were identified by RNA-seq and DAP-seq analysis, and CHIP-PCR found that the *PsnWRKY70* gene can activate homologous *WRKY6*, *WRKY18*, *WRKY22*, *WRKY22-1*, and R genes to confer higher disease resistance in *Populus*. Other members of WRKYs may also play a role in disease resistance. This study further demonstrated the role of *PsnWRKY70* in the regulatory network response to infection with *Alternaria alternata* (Fr.) Keissl in *Populus*.

## Figures and Tables

**Table 1:** Representative *PsnWRKY70* DTGs were identified by RNA-seq and DAP-seq analysis.

Gene	Description	Fold Change	*p*-Value	Peak
Potri.004G007500.1	WRKY 6	1.1051	0.0192	Chr04-461048-1
Potri.014G195100.7	LRR_8	1.3831	0.0453	Chr14-17455217-2
Potri.019G122500.2	LRR_RLK	1.5968	0.0015	Chr19-14761541-1
Potri.014G035700.1	ADR1-like 2	1.3115	0.0008	Chr14-2249744-1
Potri.011G043300.1	CBP60-C	1.7518	0.0050	Chr11-3380308-1
Potri.006G263600.1	WRKY18	1.4573	0.0089	Chr06-25984884-1
Potri.002G164400.4	WRKY22	1.1219	0.0045	Chr02-12589328-2
Potri.T001933.3	NB-ARC	1.4444	0.0281	scaffold_45-136830-2
Potri.014G090300.1.	WRKY22-1	1.0772	0.0047	Chr14-5869875-1
Potri.003G133400.1	LEA_2	1.2516	0.0002	Chr03-15146326-1
Potri.019G097300.1	LRR	3.1536	0.0385	Chr19-13468226-1
Potri.013G010700.2	SARD1	1.4677	7.7484 × 10^−6^	Chr13-680718-1

## Data Availability

All RNA-seq and DAP-seq data have been archived in the NCBI Sequence Read Archive (SRA), accession number PRJNA833248.

## Supplementary Materials

The following are available online at https://www.mdpi.com/article/10.3390/ijms23147537/s1.
